# B-Mode and Elastosonographic Evaluation to Determine the Reference Elastosonography Values for Cervical Lymph Nodes

**DOI:** 10.5402/2013/895287

**Published:** 2013-08-07

**Authors:** Aydin Kurt, Idil Gunes Tatar, Ali Ipek, Baki Hekimoglu

**Affiliations:** ^1^Diskapi Yildirim Beyazit Training and Research Hospital, Department of Radiology, Altındag 06110, Ankara, Turkey; ^2^Ataturk Training and Research Hospital, Department of Radiology, Bilkent Yolu 3. km, Cankaya 06800, Ankara, Turkey

## Abstract

*Background*. It is crucial to differentiate between reactive and malignant lymphadenopathies. Elastosonography measures the elasticity of the tissue. Having a reference value for benign lymph nodes (LNs) is important in interpretation. The purpose of this study is to determine the reference elastosonography values of cervical LNs. *Methods*. 97 LNs were evaluated by B-mode and elastosonography. Depth, length, width, length to width ratio, hilar-cortical thickness, strain ratio, and elasticity scores were measured. *Results*. In 18.6% of the cervical LNs cortical thickness was less than the hilar thickness (group A) and in 81.4% it was equal or more (group B). 69.1% of LNs showed strain ratio (SR) less than 3 (group 1) and 30.9% equal to or more than 3 (group 2). 33% of LNs displayed elasticity score (ES) 1; 30.9% ES 2; 22.7% ES 3, and 13.4% ES 4. There was a significant correlation between thickness ratios and elasticity scores (*P*: 0.011). A significant correlation was also demonstrated between SR groups and elasticity scores. *Conclusion*. A simple, reproducible, noninvasive imaging technique for diagnosis of malignant LNs is necessary. Elastosonography can aid in the differentiation of benign versus malignant cervical LNs, thus help reduce the number of unnecessary biopsies for benign processes.

## 1. Introduction

Elastosonography is an ultrasound based diagnosis which measures the elasticity or stiffness of the tissue. Compressibility of the tissue is a parameter evaluated by an elastogram and it is expressed as the change in tissue displacement as a function of its distance from the probe which is recognised as strain [1].

Among the 400–450 lymph nodes (LNs) of the human body, 60–70 are located in the head and neck area. LNs display reactive enlargement in infection and also when they are primarily or secondarily involved in malignancies of head and neck. Cervical LN metastasis may even be the first symptom in the malignant processes. It is crucial to differentiate between reactive and malignant lymphadenopathies. An important criterion is the hardness of the LN, because LNs might demonstrate increased stiffness on the elastogram if affected by a malignant process [2–4]. Having a reference value for benign LNs is important in interpretation. The purpose of this study is to determine the reference sonoelastography values of cervical LNs.

## 2. Materials and Methods

### 2.1. Patients

After obtaining the approval of the ethical committee, patients referred to the radiology department for cervical vascular Doppler ultrasonography examination from January to March 2013 were enrolled in this prospective study. Written informed consent was received from all patients. 97 cervical LNs of 89 consecutive patients (70 LNs in 64 females, 27 LNs in 25 males; mean age 54 years old; age range, 21–84 years) who were referred to our clinic for the examination of cervical vasculature were analysed by elastosonography. None of the patients had a history of malignancy. 

### 2.2. Equipment and Scanning

One radiologist with 20 years of conventional sonography and 5 years of elastosonography experience performed the examination. The scanner used was ESAOTE Gold Platform MyLab 60 (Italy) equipped with elasto software (elaxto). The probe used was 4–13 MHz linear probe.

B-mode images were first obtained for each LN. Afterwards elastosonography was performed by applying light vertical pressure followed by decompression until a good quality image was obtained. Elaxto software also made the sonographer aware of the compression degree by the help of its unique optimal compression scale for measurement. Real time B-mode and elastosonographic images were demonstrated next to each other on the screen. A region of interest (ROI) box was placed in the LN taking the adjacent subcutaneous fat tissue as the reference.

### 2.3. B Mode Evaluation

Depth, length, width, length to width ratio, hilar thickness, and cortical thickness of the LNs were measured. LNs were categorised into two groups according to their hilar/cortical thickness ratio. LNs with cortical thickness less than half of its hilar thickness were categorised as group A, and LNs with cortical thickness equal to or more than half of the hilar thickness were categorised as group B.

### 2.4. Elastographic Evaluation

**Figure 1:**
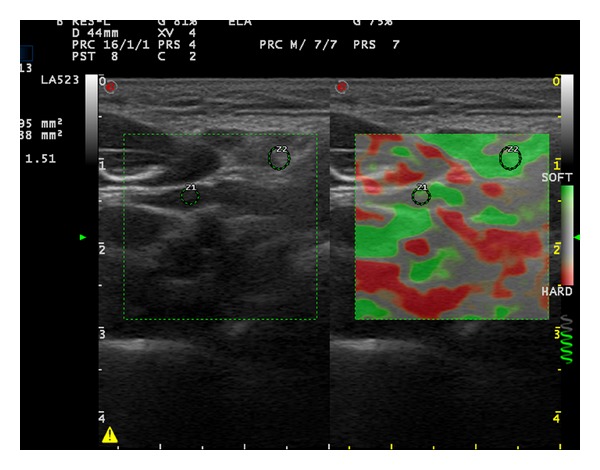
Elastosonography of the cervical lymph node on the right side of a 36-year-old male patient (long axis: 16.1 mm, short axis: 4.4 mm, cortical thickness: 1.3 mm, hilar thickness: 1.9 mm, strain ratio: 1.51, and elasticity score: 1).

**Figure 2:**
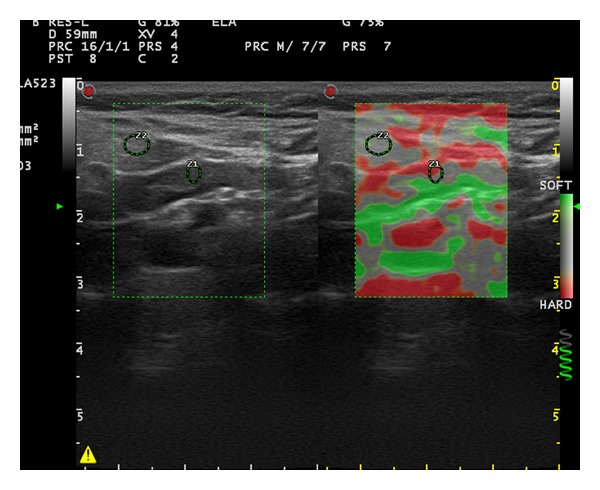
Elastosonography of the cervical lymph node on the right side of a 50-year-old female patient (long axis: 24.9 mm, short axis: 5.2 mm, cortical thickness: 3.5 mm, hilar thickness: 1.8 mm, strain ratio: 2.03, and elasticity score: 2).

**Figure 3:**
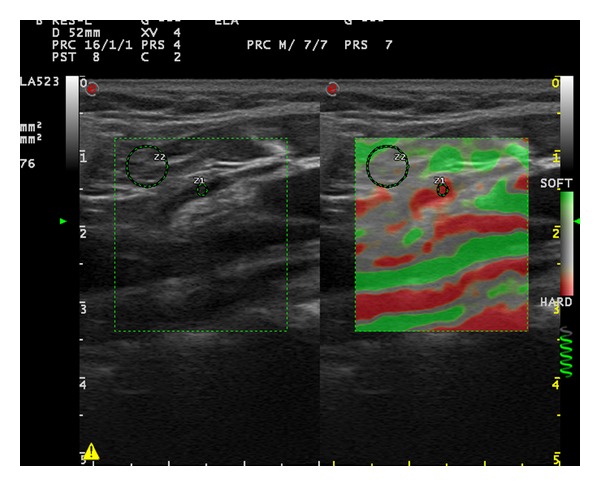
Elastosonography of the cervical lymph node on the right side of a 55-year-old female patient (long axis: 19.8 mm, short axis: 4.9 mm, cortical thickness: 1.7 mm, hilar thickness: 3.1 mm, strain ratio: 3.76, and elasticity score: 3).

**Figure 4:**
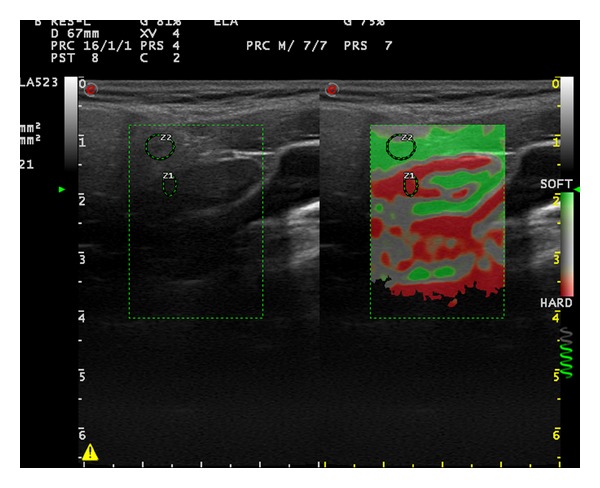
Elastosonography of the cervical lymph node on the left side of a 50-year-old female patient (long axis: 24.6 mm, short axis: 7.7 mm, cortical thickness: 2.6 mm, hilar thickness: 4.9 mm, strain ratio: 38.6, and elasticity score: 4).

First strain ratio (SR) of the LNs was measured. Afterwards, elastographic images were given 1 of 4 elasticity scores (ESs) based on the percentage of stiff areas modified from Choi et al. which was introduced for the differential diagnosis of axillary LNs in breast cancer [5]. ES 1 (Figure 1), an absent or a very small red area (i.e., stiff); ES 2 (Figure 2), small, scattered red areas, total red area < 45%; and ES 3 (Figure 3), large red area, total red area ≥ 45%; and ES 4 (Figure 4), red area occupying the entire LN, were the classifications used in the study.

## 3. Results

64 (66%) of the LNs were found on the right side and 33 (34%) were found on the left side of the neck. The mean size of the LNs was 17 mm with a range of 6–28 mm. The mean depth of the LNs was 11 mm, with a range of 5–19 mm.

18 LNs (18.6%) were categorised as group A. 79 lymph nodes (81.4%) were categorised as group B. 67 LNs (69.1%) had SR less than 3 and were categorised as group 1. 30 LNs (30.9%) with SR equal to or more than 3 were categorised as group 2. 32 LNs (33%) displayed ES 1; 30 LNs (30.9%) demonstrated ES 2; 22 LNs (22.7%) showed ES 3; 13 LNs (13.4%) had ES 4. 62 LNs (63.9%) with scores 1 and 2 were assigned as ES grade 1; 35 LNs (36.1%) having scores 3 and 4 were assigned as ES grade 2.

Statistical analysis was done with SPSS 17 programme. There was a statistically significant correlation between thickness ratio groups and elasticity score grades (*P*: 0.011). A statistically significant correlation was also demonstrated between SR groups and elasticity score grades (*P*: 0.000). 

## 4. Discussion

Elastosonography, a noninvasive imaging modality, can potentially help the differentiation of benign and malignant LNs by reducing the number of unnecessary biopsies.

In the literature, several studies have been published for the sonographic diagnosis of metastatic cervical LNs by B-mode [6–10]. B-mode criteria for evaluating superficial LNs are size, shape, the presence or the absence of the hilum, ratio of the cortex to hilum, borders, echogenicity, and homogeneity of internal structures. However no single ultrasonography criterion for malignant LNs had high sensitivity and specificity [11, 12]. Specific criteria to differentiate reactive and metastatic cervical LNs are still not well established. A simple, reproducible, noninvasive imaging technique for diagnosis of malignant LNs is necessary.

Sohn et al. investigated the B-mode criteria to differentiate metastatic LNs from benign LNs taking fine needle aspiration biopsy as the gold standard. They evaluated the diagnostic performance of each ultrasound feature (loss of fatty hilum, presence of cystic change or calcification, hyperechogenicity, and round shape). They concluded that the most accurate ultrasound criterion to differentiate metastatic from benign lymph nodes was the presence of one of these malignant ultrasound findings, excluding the loss of fatty hilum [13].

The 5-point elasticity scoring system was introduced by Itoh et al. for breast lesions [14]. Lyshchik et al. published their results in the diagnosis of cervical LN metastasis using gray-scale sonographic elastography [2]. They utilized a 4-point scale including visibility, relative brightness, margin regularity, and margin definition of the LN. They measured strain index of the LNs by comparing the absolute strain values of the lymph nodes with the absolute strain values values of the nearby muscles. Strain index greater than 1.5 was the most useful criteria in metastatic LN classification, having 98% specificity, 85% sensitivity, and 92% accuracy. 

Alam et al. used a 5-pattern color scoring system based on distribution and percentage of area with high elasticity in the cervical LNs [3]. The cut-off line for reactive versus metastatic LNs was set between patterns 2 and 3; patterns 3–5 were considered metastatic. They evaluated the sum of scores of five criteria, that is, short-axis diameter, shape, border (regular or irregular), echogenicity (homogeneous or inhomogeneous), and presence or absence of hilum for B-mode diagnosis. The cut-off line for reactive versus metastatic was set between scores 6 and 7. Scores 5 and 6 were considered reactive and scores 7–10 metastatic. Sensitivity, specificity, and accuracy of B-mode sonography were 98%, 59%, and 84%, respectively; 83%, 100%, and 89% for elastography; and 92%, 94%, and 93% for the combined evaluation. They concluded that the combination of elastography and B-mode sonography has the potential to improve the diagnosis of metastatic enlarged cervical LNs.

Arda et al. concluded that elastosonography had 93.8% sensitivity and 89.5% specificity in the differentiation of benign and malignant cervical lymph nodes [15].

In a meta-analysis of the real-time elastography for the differentiation of benign and malignant superficial LNs Ying et al. concluded that even though ES measurement had a good diagnostic accuracy, it had significant interobserver variability. Thus, a quantitative method such as strain ratio measurement was needed for analysis of elasticity of the LN. Although the accuracy of strain ratio measurement was similar to ES measurement, it improved the diagnostic sensitivity value. Their meta-analysis suggested that real-time elastography could be used as a good identification tool for malignant LNs [16]. 

Nevertheless only a limited number of studies have been carried out to determine the elasticity of various tissues. Levinson et al. evaluated the elasticity of skeletal muscles [17] and Arda et al. conducted a study to determine the elasticity values of normal soft tissue including thyroid, parotid, and submandibular glands, gastrocnemius and masseter muscles, supraspinatus and Achilles tendons, renal cortex, and pelvis, spleen, and pancreas of healthy volunteers [18]. Estimation of tissue elasticity is useful for characterization of normal tissue. To our knowledge, the normal elasticity values of cervical lymph nodes have not been studied so far. 

In our study 36.1% of the evaluated cervical LNs were considered hard, having elasticity scores 3 and 4. These LNs displayed statistically significant higher strain ratio values of more than 3. They also showed statistically significant cortical thickness ratio in favor of the cortex. Elastosonography can aid the differentiation of benign and malignant cervical LNs. The aim of elastography is not necessarily to diagnose malignancies but to help reduce the number of unnecessary biopsies for benign processes by determining soft versus hard tissues due to its high specificity [2, 3, 19].

## 5. Limitations

Elastosonography is a user dependent technique closely related to experience. Due to the shape of the neck, there is a possibility of sliding motion during compression. Problems with interpretation may occur if low-quality elastograms are obtained. The pulsations from the neighboring great vessels may also make it difficult to obtain good quality elastograms. The primary aim of this research was to determine the reference values of cervical LNs to guide elastographic studies not to establish the benign versus malignant nature of the lymph nodes. But further elastographic studies with histopathological correlations are necessary.
